# Baby-Friendly Hospital Initiative and exclusive breastfeeding during hospital stay

**DOI:** 10.11606/s1518-8787.2023057004283

**Published:** 2023-04-12

**Authors:** Mariana Pujól von Seehausen, Maria Inês Couto de Oliveira, Maria do Carmo Leal, Rosa Maria Soares Madeira Domingues, Cristiano Siqueira Boccolini

**Affiliations:** I Fundação Oswaldo Cruz Escola Nacional de Saúde Pública Sérgio Arouca Programa de Pós-Graduação em Epidemiologia em Saúde Pública Rio de Janeiro RJ Brasil Fundação Oswaldo Cruz. Escola Nacional de Saúde Pública Sérgio Arouca. Programa de Pós-Graduação em Epidemiologia em Saúde Pública. Rio de Janeiro, RJ, Brasil; II Universidade Federal Fluminense Instituto de Saúde Coletiva Departamento de Epidemiologia e Bioestatística Niterói RJ Brasil Universidade Federal Fluminense. Instituto de Saúde Coletiva. Departamento de Epidemiologia e Bioestatística. Niterói, RJ, Brasil; III Fundação Oswaldo Cruz Escola Nacional de Saúde Pública Sérgio Arouca Rio de Janeiro RJ Brasil Fundação Oswaldo Cruz. Escola Nacional de Saúde Pública Sérgio Arouca. Rio de Janeiro, RJ, Brasil; IV Fundação Oswaldo Cruz Instituto Nacional de Infectologia Evandro Chagas Rio de Janeiro RJ Brasil Fundação Oswaldo Cruz. Instituto Nacional de Infectologia Evandro Chagas. Rio de Janeiro, RJ, Brasil; V Fundação Oswaldo Cruz Instituto de Comunicação e Informação Científica e Tecnológica em Saúde Laboratório de Informação em Saúde Rio de Janeiro RJ Brasil Fundação Oswaldo Cruz. Instituto de Comunicação e Informação Científica e Tecnológica em Saúde. Laboratório de Informação em Saúde. Rio de Janeiro, RJ, Brasil

**Keywords:** Breast Feeding, Hospitals, Maternity, Postpartum Period, Cross-Sectional Studies

## Abstract

**OBJECTIVE:**

To estimate the prevalence of exclusive breastfeeding during maternity hospital stay (outcome) and to analyze the association between delivery in a Baby-Friendly Hospital (BFH) and the outcome. The hypothesis is that accreditation to this program improves exclusive breastfeeding during maternity hospital stay. Exclusive breastfeeding is essential in reducing neonatal morbidity and mortality.

**METHODS:**

This study is based on secondary data collected by the “Birth in Brazil: National Survey into Labour and Birth”, a population-based study, conducted with 21,086 postpartum women, from February 1, 2011, to October 31, 2012, in 266 hospitals from all five Brazilian regions. Face-to-face interviews were conducted mostly within the first 24 hours after birth, regarding individual and gestational characteristics, prenatal care, delivery, newborn’s characteristics, and breastfeeding at birth. A theoretical model was created, allocating the exposure variables in three levels based on their proximity to the outcome. This hierarchical conceptual model was applied to perform a multiple logistic regression (with 95%CI and p < 0.05).

**RESULTS:**

In this study, 76.0% of the babies were exclusively breastfed from birth until the interview. Babies born in public (AOR = 1.73; 95%CI: 1.10–2.87), mixed (AOR = 2.48; 95%CI: 1.35–4.53) and private (AOR = 5.54; 95%CI: 2.38–12.45) BFHs were more likely to be exclusively breastfed during maternity hospital stay than those born in non–BFHs, as well as those born by vaginal birth (AOR = 2.16; 95%CI: 1.79–2.61), with adolescent mothers (AOR = 1.83; 95%CI: 1.47–2.26) or adults up to 34 years old (AOR =1 .31; 95%CI: 1.13–1.52), primiparous women (AOR = 1.51; 95%CI: 1.34–1.70), and mothers living in the Northern region of Brazil (AOR = 1.99; 95%CI: 1.14–3.49).

**CONCLUSIONS:**

The Baby-Friendly Hospital Initiative promotes exclusive breastfeeding during hospital stay regarding individual and hospital differences.

## INTRODUCTION

Exclusive breastfeeding is essential in reducing neonatal morbidity and mortality: the chances of death resulting from infection are higher in newborns who receive other liquid or food rather than breast milk during this period. Exclusively breastfed infants have a lower risk of developing sepsis, diarrhea, and respiratory infections. Moreover, the later breastfeeding begins, the greater the chances of neonatal death caused by infections^1,2^.

Multiple mechanisms can explain the protective effect of breast milk. It contains several bioactive immunological factors, such as immunoglobulin A, cytokines, and lactoferrin. Oligosaccharides, secreted in abundance in colostrum and mature breast milk, act as a substrate for bacteria from the healthy intestinal microbiota and prevent pathogens’ attachment to the baby’s mucosal membranes, reducing the risk of viral, bacterial, and protozoal infections. Oligosaccharides can also modulate the immune and epithelial responses and decrease the risk of necrotizing enterocolitis^3^.

Aiming to promote, protect, and support breastfeeding, the United Nations Children’s Fund (UNICEF) and the World Health Organization (WHO) released in 1991 the Baby-Friendly Hospital Initiative (BFHI), adopted in Brazil since 1992. The Initiative is also part of the Global Strategy for Infant and Young Child Feeding, created in 2002 by UNICEF and the WHO. The Ten Steps to Successful Breastfeeding, a package of procedures that facilities providing maternity and newborn services should support, is the basis of this Initiative. The sixth step states: “Do not provide breastfed newborns any food or fluids other than breast milk unless medically indicated”, thus these maternities are committed to promote exclusive breastfeeding^4^.

Using data from a populational based survey, we aimed to estimate the prevalence of exclusive breastfeeding during hospital stay and to analyze the association between delivery in a Baby-Friendly Hospital (BFH) and exclusive breastfeeding. The hypothesis is that accreditation to this program improves exclusive breastfeeding during stay in maternity hospitals.

## METHODS

This is a study of national coverage and hospital basis, founded on secondary data from the research called “Birth in Brazil: National Survey into Labour and Birth” (“*Nascer no Brasil: Inquérito Nacional Sobre Parto e Nascimento”*). Its complex sample represented all births occurring in hospitals with more than 500 births/year in Brazil (corresponding to 78.6% of all hospital births). The research was coordinated by Fundação Oswaldo Cruz, after approval by the Institutional Review Board of ENSP/FIOCRUZ and was conducted between February 2011 and October 2012^5^.

The sampling process was performed in three stages. First, the sample was stratified into three levels: by the Brazilian region (North, Northeast, Midwest, Southeast, and South), by location (capital or non-capital city), and by hospital funding source (public, private or mixed), thus obtaining 30 strata. Within each stratum, hospitals were selected by probability proportional to size, defined by the number of live births in the hospital according to the 2007 Brazilian Birth Certificate System (SINASC, *Sistema de Informações Sobre Nascidos Vivos*) data. In the second stage, the inverse sampling method was used to determine the number of days required to evaluate 90 puerperal women per hospital. To account for the difference in the number of live births on weekends and workdays, the field researchers stood at least seven days in each hospital to ensure representative samples’ recruitment. In the third stage, the eligible women were selected on each day of fieldwork. Due to early discharge, refusals or losses, mothers were replaced by others in the postpartum period at the same hospital^6^.

Hospitals that had 500 or more deliveries in 2007 were considered eligible. In each hospital, women who gave birth to newborns of any gestational age or weight or stillborn babies weighing ≥ 500g or gestational age ≥ to 22 weeks were considered eligible by the original research project. The sample size in each stratum was calculated based on the proportion of cesarean sections in Brazil in 2007 of 46.6%, with a significance level of 5% and 95% power, to detect differences of at least 14% between public, mixed, and private hospitals. At least five hospitals were selected per stratum, and 90 postpartum women per hospital, totaling 23,894 postpartum women interviewed^5^.

An electronic questionnaire was applied by trained field researchers, within at least 6h after a vaginal delivery and 12h after a cesarean section, with questions regarding individual and gestational characteristics, prenatal care, delivery, newborn’s characteristics and breastfeeding at birth. Another questionnaire was applied to the manager of each maternity hospital regarding the hospital characteristics. More detailed information on fieldwork can be found in a previous publication^5^.

In the present study, exclusive breastfeeding during postpartum hospital stay (outcome) was investigated. The outcome was based on a question asked to the mothers: “(Here) at the hospital, did (baby’s name) receive any milk or liquid other than your breast milk?” which was dichotomously categorized (yes, no). Based on the conditions that may impede breastfeeding, we established the following exclusion criteria: mothers with HIV-positive serology^7^, maternal near-miss diagnosis^8^; fetal or neonatal deaths; infants below 34 weeks of gestational age; and infants with severe malformations or a neonatal near miss diagnosis^9^. Moreover, second and third twins were excluded, resulting in a final sample of 21,086 mothers and their respective babies.

The primary exposure variable consisted of a combination of the variables “Baby-Friendly Hospital delivery” (dichotomously categorized: no or yes, or undergoing the accreditation process) and “hospital funding source” (categorized as public, private or mixed, with the latter category referring to private hospitals that have a contract with the Brazilian Unified Health System), both obtained from the questionnaire applied to the manager of each maternity hospital. The variables “BFH delivery” and “hospital funding source” were combined, since a high correlation (p < 0.001) between them in the exploratory data analysis was observed. Thus, the primary exposure variable was created, called “place of delivery”, and categorized into six possibilities: public BFH, private BFH, mixed BFH, public non-BFH, private non-BFH, and mixed non-BFH. The first three categories refer to hospitals that receive public, private, or mixed-funding and are accredited to or undergoing the process of being certified to the Baby-Friendly Hospital Initiative.

Based on a literature review^10^ and according to the assumptions of the hierarchical organization of variables proposed by Victora et al.^13^, a theoretical-conceptual model was created, in which the exposure variables were allocated at three levels (distal, intermediate, and proximal) based on the proximity between each variable and the outcome. Maternal characteristics (distal level): age, educational level, parity, and region of residence; and characteristics of prenatal care (intermediate level): prenatal funding, information on breastfeeding, and prenatal adequacy; as well as characteristics of delivery and infant care (proximal level): place of delivery, type of delivery, obstetric risk, postpartum companion, neonatal ICU stay, and gestational age were investigated. The time (in hours) between delivery and the interview was also included in the model, as it varied between 6h and 120h.

The variable “information about breastfeeding” refers to having received information, during prenatal care, about the importance of early breastfeeding initiation. The variable “prenatal adequacy” was measured by the Kotelchuck Index, which evaluates the number of prenatal consultations based on the month when prenatal care started^14^. For the variables related to prenatal care, “prenatal funding”, “information on breastfeeding”, and “prenatal adequacy”, women who did not receive prenatal care (n = 205; 0.97% of the sample) were excluded. The variable “type of delivery” comprised three possibilities: vaginal, antepartum cesarean section, and intrapartum cesarean section. Regarding the variable “obstetric risk”, mothers who had one or more of the following conditions or diseases at high risk were considered: hypertensive syndromes (gestational hypertension, chronic hypertension, preeclampsia, eclampsia, HELLP syndrome), pre-gestational diabetes, gestational diabetes, severe chronic diseases, infection at hospital admission (including urinary tract infection and other severe infections, such as chorioamnionitis and pneumonia), premature placental abruption (PPA), placenta previa, and intrauterine growth restriction (IUGR)^14^.

Initially, the frequency of all exposure variables and the study outcome were analyzed. The complex design of the sample for all further analysis^6^ was considered. A bivariate analysis was performed between each confounding variable and the outcome, using simple logistic models to obtain unadjusted odds ratios (OR) and their respective 95% confidence intervals (95%CI). For the multiple logistic regression, performed subsequently, the hierarchical conceptual model was applied in three stages. First, at the same time, all distal variables associated with the outcome in the unadjusted analyses were estimated. We removed the variables that did not reach statistical significance at this stage (*p* > 0.05). Second, we entered the previously selected intermediate variables, and the ones that did not reach a *p*-value < 0.05 were removed, preserving all distal variables chosen in the first stage. By the last stage, the proximal variables were estimated and only those with p < 0.05 remained in the model, preserving all distal and intermediate variables selected in the first and second stages. The data was analyzed by using the SPSS statistical package.

## RESULTS

Almost one fifth were adolescent mothers, and less than half have completed high school. Three-quarters of the mothers received prenatal care via the Brazilian Unified Health System (SUS). Regarding delivery, 23.5% of the mothers gave birth in accredited public hospitals or public hospitals which were undergoing the process of accreditation to the Baby-Friendly Hospital Initiative. Only 0.8% of the mothers delivered in private accredited hospitals. Regarding characteristics of birth delivery, 42.8% underwent antepartum cesarean sections, and 6.5% had preterm babies (gestational age between 34 0/7 and 36 6/7 weeks). Considering the babies, 76.0% (72.9%-79.0%) were exclusively breastfed during hospital stay, from delivery until the time of the interview (Table 1).

**Table 1:** Distribution of maternal, newborn, and hospital characteristics. Brazil, 2011–2012.

Variables	n^a^	%^b^	95%CI^b^
Maternal age			
12–19 years old	3,839	19.2	18.1–20.5
20–34 years old	14,983	70.8	69.6–72.0
35 years or older	2,264	9.9	9.1–10.8
Maternal education level			
Complete Elementary School	10,343	52.2	49.9–54.4
High School or higher	10,743	47.8	45.6–50.1
Parity			
Primiparous	9,826	46.6	45.2–48.0
Multiparous	11,260	53.4	52.0–54.8
Region of Residence			
North	2,593	9.8	8.8–10.8
Northeast	5,429	29.0	26.6–31.4
Southeast	6,992	42.3	39.3–45.3
South	3,575	12.4	11.2–13.7
Midwest	2,497	6.6	5.5–7.9
Prenatal Funding^c^			
Public service	14,369	74.7	73.2–76.2
Private service	6,467	25.3	23.8–26.8
Prenatal breastfeeding information^c^			
Yes	13,901	64.1	62.1–66.1
No	6,889	34.8	32.9–36.8
Prenatal adequacy^c^			
Yes	13,885	65.3	64.0–66.6
No	6,794	34.7	33.4–36.0
Place of delivery^d^			
Public BFH	4,007	23.5	19.4–28.2
Public non–BFH	3,283	16.3	12.7–20.6
Mixed BFH	3,350	15.5	12.0–19.9
Mixed non–BFH	5,907	29.9	25.5–34.8
Private BFH	237	0.8	0.2–2.8
Private non–BFH	4,302	14.0	12.6–15.5
Type of delivery			
Vaginal	10,061	48.9	45.8–52.0
Intrapartum caesarean section	1,727	8.3	7.1–9.7
Antepartum caesarean section	9,298	42.8	40.2–45.5
Obstetric risk			
High	4,437	21.8	20.6–23.1
Low	16,649	78.2	76.9–79.4
Postpartum companion			
Yes	13,312	61.5	57.6–65.3
No	7,774	38.5	34.7–42.4
Gestational age (weeks)			
34 0/7 to 36 6/7	1,278	6.5	5.7– 7.3
37 0/7 or more	19,808	93.5	92.7–94.3
Newborn admission at neonatal ICU			
Yes	187	0.9	0.7–1.2
No	20,899	99.1	98.8–99.3
Exclusive breastfeeding during hospital stay			
Yes	16,025	76.0	72.9–79.0
No	5,061	24.0	21.0–27.1

BFH: Baby-Friendly Hospital; ICU: intensive care units.

^a^ Final sample of mothers who answered the “*Nascer Brasil*” survey questionnaire in 2011 and met the study criteria - cases without correction by sample weight.

^b^ Frequency and 95% confidence interval (95%CI) of the valid final sample, considering the complex sample design.

^c^ Excluding women who did not receive prenatal care (n = 205).

^d^ Based on information collected from the hospital manager.

The median of hours between delivery and the interview was 20.5 hours. The prevalence of exclusive breastfeeding during hospital stay was lower in non-BFHs and higher in accredited maternity hospitals or those undergoing the accreditation process, whether private, mixed, or public. Among the non-accredited ones, the public and mixed showed a higher prevalence of the outcome. In the bivariate analysis, were associated with the outcome the distal variables: maternal age, educational level, parity, and region of residence; the intermediate variables: prenatal funding and adequacy; and the proximal variables: place of delivery, type of delivery, obstetric risk, gestational age, admission at the neonatal ICU, as well as the time between delivery and the interview (Table 2).

**Table 2:** Prevalence of exclusive breastfeeding during hospital stay according to maternal, newborn, and hospital characteristics. Brazil, 2011–2012.

Variables	Prevalence^a^	95%CI^a^	Crude OR^b^	95%CI^b^
Maternal age				
12–19 years old	82.4	79.3–85.1	2.20	1.83–2.65
20–34 years old	75.4	72.1–78.5	1.44	1.26–1.65
35 years or older	68.0	63.5–72.3	1.00	
Maternal educational level				
Complete Elementary School	81.4	78.4–84.0	1.85	1.60–2.14
Complete High School or higher	70.2	66.3–73.8	1.00	
Parity				
Primiparous	79.4	76.3–82.3	1.50	1.35–1.66
Multiparous	72.1	68.6–75.4	1.00	
Region of residence				
North	85.6	78.7–90.6	2.39	1.37–4.16
Northeast	78.0	73.0–82.4	1.43	0.96–2.11
2Southeast	71.4	65.3–76.8	1.00	
South	79.9	70.7–86.7	1.59	0.90–2.82
Midwest	74.2	65.5–81.3	1.15	0.70–1.91
Prenatal Funding^c^				
Public service	80.6	77.4–83.7	2.48	1.94–3.18
2Private service	63.5	56.9–67.8	1.00	
Prenatal breastfeeding information^c^				
Yes	76.5	72.4–80.1	1.07	0.91–1.27
No	75.2	69.8–79.9	1.00	
Prenatal adequacy^c^				
Yes	73.7	70.3–76.8	0.67	0.59–0.78
No	80.6	77.2–83.5	1.00	
Place of delivery^d^				
Public BFH	82.8	77.0–87.4	3.13	1.93–5.09
Public Non–BFH	78.0	70.4–82.5	3.06	1.82–5.17
Mixed BFH	85.3	79.0–90.0	4.24	2.43–7.39
Mixed Non–BFH	72.4	65.2–78.6	1.91	1.18–3.09
Private BFH	88.3	80.6–93.2	5.50	2.75–11.01
Private Non–BFH	57.9	49.3–66.0	1.00	
Type of delivery				
Vaginal	85.7	82.8–88.2	3.09	2.55–3.74
Intrapartum caesarean section	69.4	64.0–74.3	1.16	0.94–1.43
Antepartum caesarean section	66.1	61.8–70.1	1.00	
Obstetric risk				
High	67.3	62.7–71.5	0.56	0.48–0.65
Low	78.5	75.4–81.3	1.00	
Postpartum companion				
Yes	74.9	71.4–78.2	0.85	0.66–1.09
No	77.9	73.4–81.9	1.00	
Gestational age (weeks)				
34 0/7 to 36 6/7	63.9	58.6–68.8	0.53	0.45–0.63
37 0/7 or more	76.9	73.7–79.3	1.00	
Newborn admission at neonatal ICU				
Yes	53.3	39.8– 66.4	0.36	0.21–0.61
No	76.2	73.0–79.1	1.00	
Time between delivery and interview (in hours)	–	–	0.97	0.96–0.98
Total	76.0	72.9–79.0		

BFH: Baby-Friendly Hospital; ICU: intensive care units.

^a^ Prevalence of exclusive breastfeeding and 95% confidence interval (95%CI) of the valid final sample, considering its complex design.

^b^ Unadjusted odds ratio (OR) and 95% confidence interval (95%CI), considering the complex sample design.

^c^ Excluding women who did not receive prenatal care (n = 205).

^d^ Based on information collected from the hospital manager.

In the adjusted model, adolescent and adult mothers up to 34 years old were more likely to have their children exclusively breastfed during hospital stay, as well as primiparous women, those living in the Northern region, mothers who had their babies in public, mixed or private Baby-Friendly Hospitals, and those who had a vaginal birth. Mothers classified as having high obstetric risk, with preterm babies or admitted to a neonatal ICU were less likely to exclusively breastfeed their babies during hospital stay. For each additional hour between birth and the interview, the chance of exclusive breastfeeding in the hospital decreased by 3% (Table 3).

**Table 3:** Factors associated with exclusive breastfeeding during hospital stay. Brazil, 2011–2012.

Variables	Adjusted OR^a^	95%CI^a^
Maternal age		
12–19 years old	1.83	1.47–2.26
20–34 years old	1.31	1.13–1.52
35 years or older	1.00	
Maternal educational level		
Complete Elementary School	1.10	0.93–1.25
Complete High School or higher	1.00	
Parity		
Primiparous	1.51	1.34–1.70
Multiparous	1.00	
Region		
North	1.99	1.14–3.49
Northeast	1.32	0.87–2.01
Southeast	1.00	
South	1.56	0.85–2.89
Midwest	1.16	0.69–1.95
Place of delivery^b^		
Public BFH	1.73	1.10–2.87
Public non–BFH	1.65	0.94–2.91
Mixed BFH	2.48	1.35–4.53
Mixed non–BFH	1.30	0.77–2.18
2Private BFH	5.44	2.38–12.45
Private non–BFH	1.00	
Type of delivery		
Vaginal	2.16	1.79–2.61
Intrapartum caesarean section	0.96	0.80–1.16
Antepartum caesarean section	1.00	
Obstetric risk		
High	0.73	0.63–0.85
Low	1.00	
Gestational age (weeks)		
34 0/7 to 36 6/7	0.49	0.41–0.59
37 0/7 or more	1.00	
Newborn admission at neonatal ICU		
Yes	0.49	0.28–0.88
No	1.00	
Time between delivery and interview		
In hours	0.97	0.96–0.98

BFH: Baby-Friendly Hospital; ICU: intensive care units.

^a^ Adjusted odds ratio (OR) and 95% confidence interval (95%CI), obtained from the logistic regression model, considering the complex sample design.

^b^ Based on information collected from the hospital manager.

## DISCUSSION

Approximately three-quarters of Brazilian mothers exclusively breastfed their babies during maternity hospital stay in 2011 and 2012. The only Brazilian study that also investigated this outcome found a high prevalence of supplementation with infant formula in a public hospital in the city of Rio de Janeiro (49.8%)^15^. In other countries, exclusive breastfeeding during hospital stay ranged from 19.1% in five Greek private and public hospitals^12^ to 40.5% in 17 Italian national health service hospitals^16^.

Mothers who gave birth in accredited Baby-Friendly Hospitals or in hospitals undergoing the accreditation process were more likely to exclusively breastfeed during hospital stay, whether they were public, mixed, or private hospitals. These results show the association between the BFHI and better exclusive breastfeeding rates. Brazilian Baby-Friendly Hospitals are reassessed triennially by external evaluators and annually by self-monitoring, to maintain their quality care in promoting, protecting, and supporting breastfeeding^17^.

Although the results were positive for BFHs in general, private and mixed-funded hospitals had even higher chances of exclusive breastfeeding practice than public hospitals. The superior performance of the mixed-funded BFHs over the public-funded ones might relate to extra incentives: although both receive incentives once they become accredited to the Initiative, the payment mechanisms are different. The government’s payment of public-funded hospitals is set annually, and the budget is allocated in advance, with little local managerial autonomy to reallocate resources. Although this makes it easier to control the total spending, it is not performance-related and does little to promote service provision efficiency^18^.

The payment of mixed-funded hospitals relies on reimbursement for predefined and standardized sets of services, where the hospital receives a fixed amount per hospitalization according to the group of medical procedures. While most mixed hospitals rely almost exclusively on the funds’ transfer by the governmental resources, there is a large gap between the actual costs of services and the reimbursement values paid by the SUS for most hospital procedures. This gap has been partially counterbalanced by additional payments^18^, such as those received by Baby-Friendly Hospitals, of which delivery care and newborn care procedures at the delivery room show increments that range from 2.5% to 17%^19^.

Although private non-BFHs had the lowest prevalence of exclusive breastfeeding, when the BFHI was present in the private sector, it significantly increased the chances of the outcome by more than five times. The frequency of mothers giving birth at private BFHs was less than 1%, indicating a low adherence of the private sector to the Initiative, which may relate to the Brazilian Ministry of Health requirement for additional BFHI accreditation criteria, including the reduction of invasive birth procedures, among which are cesarean sections^19^. This criterion might be challenging to be met by the private sector, as the prevalence of cesarean sections in this sector was around 90% in 2011-2012^20^, indicating the need to reduce this obstetric procedure and increase private accreditation maternity hospitals to the Initiative. The positive result observed in the few private BFHs might relate to the joint adoption of good prenatal, childbirth, and postpartum care practices^17^.

Other studies identified accreditation to the Initiative as a protective factor for breastfeeding in the first hour of life, a breastfeeding outcome in the hospital environment^21,22^.

The adherence of maternity hospitals to practices that promote, protect, and support breastfeeding is especially important, since in a hospital setting mothers have little or no decision-making power about breastfeeding and depend on the adopted institutional practices^23^. Therefore, the results found herein corroborate the importance of the Baby-Friendly Hospital Initiative for breastfeeding practices in maternity hospitals, whether public, mixed or privately funded.

Women who gave birth by vaginal delivery were twice as likely to exclusively breastfeed during hospital stay. Studies evaluating exclusive breastfeeding at hospital discharge in Greece^12^, and China^24^ also observed a similar result. In Brazil, cesarean section has also been a risk factor for non-breastfeeding in the first hour of life^22,23^. However, with adequate support, delivery by a cesarean section should not be a barrier to the opportune start of breastfeeding^25^.

Preterm newborns had half the chance of being exclusively breastfed during hospital stay. However, we included only preterm newborns with more than 34 0/7 weeks of gestation in this study, because the 34-week gestation mark is the point in which the suck, swallow, and breath coordination reflexes start to develop, and providing breastfeeding support can help mothers of preterm infants to be less anxious and practice techniques that facilitate emptying of the breast and production of an adequate milk supply^26^.

Babies admitted to a neonatal ICU also had half the chance of being exclusively breastfed during hospitalization. Recently, the BFHI has been adapted to the NICUs (Neo-BFHI), and a Spanish study showed that hospitals with BFHI accreditation or in the process accreditation had better implementation of practices to support newborns admitted to a neonatal ICU, such as stimulation provided by milking the breast to maintain lactation^27^. In Londrina, a city in southern Brazil, the prevalence of exclusive breastfeeding in newborn infants admitted to a neonatal ICU increased from 1.9% to 41.7% after BFHI implementation in a maternity hospital^28^, indicating that breastfeeding exclusively newborns in a neonatal unit is a challenge.

Compared to those at low risk, mothers in high obstetric risk were 27% less likely to breastfeed exclusively during hospital stay. Studies that investigated the association between obstetric risk and breastfeeding outcomes were not found, indicating the invisibility of these women in the studies, possibly due to difficulties in characterizing and defining obstetric risk or because their conditions or illnesses were included in the adopted exclusion criteria. Among all mothers with high obstetric risk, 67.3% exclusively breastfed in the maternity hospital, which, in absolute terms, represents a prevalence only 10.2% lower than women classified as “low risk”, indicating that exclusive breastfeeding is a possibility that should be assessed individually.

Three distal factors were associated with the outcome. Mothers aged 35 years or older were less likely to exclusively breastfed in the maternity hospital. This result has also been documented regarding breastfeeding in the first hour of life and is attributed to a possible cohort effect, since older mothers have been less exposed to the increasing practice of breastfeeding in Brazil. A similar result was found in four Ontario hospitals, where maternal age greater than 34 years was associated to lower rates of exclusive breastfeeding during hospital stay^29^. Besides, the chances of the outcome were higher for mothers living in the Northern region of the country, which may relate to the indigenous culture’s influence in this region^22^. Primiparous mothers showed a higher chance of the outcome, similarly to findings of an American study^11^, possibly due to extra support provided to first-time mothers during maternity hospital stay. However, multiparous mothers should not be overlooked, as prior experiences may not contribute to exclusive breastfeeding.

The chance of exclusive breastfeeding in the maternity hospital decreased by 3% every hour since birth, i.e., the longer the mother and her newborn stood at the hospital, the greater the chances that infant formulas would be given to the baby. Most infant formulas given at the maternity hospital are unnecessary and are not clinically justified^30^.

The proximal variables related to delivery and baby care were those most strongly associated with the outcome, reinforcing the hypothesis that hospital practices prevail over maternal individual choices^23^.

The present study has limitations. Our analysis considered data obtained via interviews from 6h to 120h after delivery. Therefore, if the interviews had been conducted at hospital discharge, probably we would have found a lower prevalence of exclusive breastfeeding, since the longer the time between delivery and the interview, the lower the chances that the mother was practicing exclusive breastfeeding. The assessed outcome was measured by asking a question to the mother, who may not have witnessed or been informed about infant formula being administered to the newborn, thus possibly creating an information bias, which also may lead to the outcome overestimation. Moreover, outcome information was collected at a single moment. To better determine the outcome, we recommend to continually assess the breastfeeding status from birth up to hospital discharge, triangulating information from medical records, nutrition service, and mothers.

We conclude that the Baby-Friendly Hospital Initiative promotes exclusive breastfeeding in public, mixed, and private funded maternity hospitals. Therefore, we recommend making investments to strengthen this Initiative in Brazil, expanding its implementation also in the private sector maternity hospitals, where the number of hospitals involved with the Initiative is still scarce.

## References

[1] Khan J, Vesel L, Bahl R, Martines JC (2015). Timing of breastfeeding initiation and exclusivity of breastfeeding during the first month of life: effects on neonatal mortality and morbidity - a systematic review and meta-analysis. Matern Child Health J.

[2] Boccolini CS, Carvalho ML, Oliveira MI, Pérez-Escamilla R (2013). Breastfeeding during the first hour of life and neonatal mortality. J Pediatr (Rio J).

[3] Nolan LS, Parks OB, Good M (2019). A review of the immunomodulating components of maternal breast milk and protection against necrotizing enterocolitis. Nutrients.

[4] World Health Organization, UNICEF (2020). Ten steps to successful breastfeeding.

[5] Leal MC, Silva AAM, Dias MAB, Gama SGN, Rattner D, Moreira ME (2012). Birth in Brazil: national survey into labour and birth. Reprod Health.

[6] Vasconcellos MTL, Silva PL, Pereira AP, Schilithz AO, Souza PR, Szwarcwald CL (2014). Sampling design for the Birth in Brazil: National Survey into Labor and Birth. Cad Saude Publica.

[7] Domingues RM, Szwarcwald CL, Souza PR, Leal MC (2015). Prenatal testing and prevalence of HIV infection during pregnancy: data from the “Birth in Brazil” study, a national hospital-based study. BMC Infect Dis.

[8] Dias MAB, Domingues RMSM, Schilithz AOC, Nakamura-Pereira M, Diniz CSG, Brum IR (2014). Incidence of maternal near miss in hospital child birth and postpartum: data from the Birth in Brazil study. Cad Saude Publica.

[9] Silva AAM, Leite AJM, Lamy ZC, Moreira MEL, Gurgel RQ, Cunha AJLA, Leal MC (2014). Neonatal near miss in the Birth in Brazil survey. Cad Saude Publica.

[10] Bramson L, Lee JW, Moore E, Montgomery S, Neish C, Bahjri K, Melcher CL (2010). Effect of early skin-to-skin mother-infant contact during the first 3 hours following birth on exclusive breastfeeding during the maternity hospital stay. J Hum Lact.

[11] Pierro J, Abulaimoun B, Roth P, Blau J (2016). Factors associated with supplemental formula feeding of breastfeeding infants during postpartum hospital stay. Breastfeed Med.

[12] Pechlivani F, Vassilakou T, Sarafidou J, Zachou T, Anastasiou CA, Sidossis LS (2005). Prevalence and determinants of exclusive breastfeeding during hospital stay in the area of Athens, Greece. Acta Paediatr.

[13] Victora CG, Huttly SR, Fuchs SC, Olinto MT (1997). The role of conceptual frameworks in epidemiological analysis: a hierarchical approach. Int J Epidemiol.

[14] Leal MC, Esteves-Pereira AP, Nakamura-Pereira M, Torres JA, Theme-Filha M, Domingues RMSM (2016). Prevalence and risk factors related to preterm birth in Brazil. Reprod Health.

[15] Lopes FO, Oliveira MIC, Brito AS, Fonseca VM (2013). Factors associated with the use of supplements among newborns in communal wards in Rio de Janeiro, 2009. Cienc Saude Colet.

[16] Asole S, Spinelli A, Antinucci LE, Di Lallo D (2009). Effect of hospital practices on breastfeeding: a survey in the Italian Region of Lazio. J Hum Lact.

[17] Araújo RG, Fonseca VM, Oliveira MIC, Ramos EG (2019). External evaluation and self-monitoring of the Baby-friendly Hospital Initiative’s maternity hospitals in Brazil. Int Breastfeed J.

[18] La Forgia GM, Couttolenc BF (2009). Desempenho hospitalar brasileiro: em busca da excelência.

[19] Ministério da Saúde (BR) (2014). Portaria Nº 1.153 de 22 de maio de 2014. Redefine os critérios de habilitação da Iniciativa Hospital Amigo da Criança (BFHI), como estratégia de promoção, proteção e apoio ao aleitamento materno e à saúde integral da criança e da mulher, no âmbito do Sistema Único de Saúde (SUS).

[20] Domingues RMSM, Dias MAB, Nakamura-Pereira M, Torres JA, d’Orsi E, Pereira APE (2014). Process of decision-making regarding the mode of birth in Brazil: from the initial preference of women to the final mode of birth. Cad Saude Publica.

[21] Esteves TMB, Daumas RP, Oliveira MIC, Andrade CAF, Leite IC (2014). Factors associated to breastfeeding in the first hour of life: systematic review. Rev Saude Publica.

[22] Carvalho ML, Boccolini CS, Oliveira MIC, Leal MC (2016). The baby-friendly hospital initiative and breastfeeding at birth in Brazil: a cross sectional study. Reprod Health.

[23] Boccolini CS, Carvalho ML, Oliveira MIC, Vasconcellos AGG (2011). Factors associated with breastfeeding in the first hour of life. Rev Saude Publica.

[24] Qiu L, Zhao Y, Binns CW, Lee AH, Xie X (2009). Initiation of breastfeeding and prevalence of exclusive breastfeeding at hospital discharge in urban, suburban and rural areas of Zhejiang China. Int Breastfeed J.

[25] Rollins NC, Bhandari N, Hajeebhoy N, Horton S, Lutter CK, Martines JC (2016). Why invest, and what it will take to improve breastfeeding practices?. Lancet.

[26] Cartwright J, Atz T, Newman S, Mueller M, Demirci JR (2017). Integrative review of interventions to promote breastfeeding in the late preterm infant. J Obstet Gynecol Neonatal Nurs.

[27] Alonso-Díaz C, Utrera-Torres I, Alba-Romero C, Flores-Antón B, Lora-Pablos D, Pallás-Alonso CR (2016). Breastfeeding support in Spanish neonatal intensive care units and the Baby-Friendly Hospital Initiative: a national survey. J Hum Lact.

[28] Vannuchi MTO, Monteiro CA, Rea MF, Andrade SM, Matsuo T (2004). The Baby-Friendly Hospital Initiative and breastfeeding in a neonatal unit. Rev Saude Publica.

[29] Finkelstein SA, Keely E, Feig DS, Tu X, Yasseen AS, Walker M (2013). Breastfeeding in women with diabetes: lower rates despite greater rewards. A population-based study. Diabet Med.

[30] Meirelles CAB, Oliveira MIC, Mello RR, Varela MAB, Fonseca VM (2008). Justifications for formula supplementation in low-risk newborns at a Baby-Friendly Hospital. Cad Saude Publica.

